# Immediate and localized effect of Kinesio tape on the hand grip strength of sedentary female adults

**DOI:** 10.25122/jml-2023-0333

**Published:** 2023-12

**Authors:** Mariam Ameer, Ammar Al Abbad, Arun Vijay Subbarayalu, Areej Alsharari, Rawan AlRuwaili, Saylah AlFuhigi, Nadia Hmdan, Amjad Alshammari, Ghala Alhuthayl

**Affiliations:** 1 Department of Physical Therapy and Health Rehabilitation, College of Applied Medical Sciences, Jouf University, Al-Jouf, Saudi Arabia; 2 Department of Biomechanics, Faculty of Physical Therapy, Cairo University, Cairo, Egypt; 3 Deanship of Quality and Academic Accreditation, Department of Physical Therapy, Imam Abdulrahman Bin Faisal University, Dammam, Saudi Arabia

**Keywords:** hand grip strength, Kinesio tape, hand-held dynamometer, healthy female adult

## Abstract

The current study aimed to assess the immediate and localized effect of Kinesio Taping (KT) on hand grip strength. A cross-sectional study was conducted on 60 sedentary female university students (aged 18-23) divided into two groups of 30 subjects each. The experimental group received KT with 50% tension of the tape on the forearm and 100% tension on the hand, and the control group received a placebo application of KT (KT without tension on the hand and forearm). Hand grip strength was assessed before and immediately after applying KT using a hand-held dynamometer with a one-minute rest between trials. The experimental group detected a significant improvement in hand grip strength during the post-intervention stage compared to the control group (mean difference 9.72 Lbs; 95% CI, -12.90 to -6.54; P<0.05) with a medium effect size. In addition, a significant improvement in handgrip strength was observed between pre-intervention and post-intervention in the experimental group (mean difference 6.5 Lbs.; 95% CI, -7.58 to -5.42; P<0.05) with a high effect size. However, the control group failed to show significant improvement in handgrip strength between pre-and post-intervention (P=0.666). KT application on the hand and forearm immediately augmented the hand grip strength of the dominant hand in sedentary female university students.

## INTRODUCTION

The Kinesio Taping (KT) technique was developed by Kenzo Kase in 1996, which comprises the direct use of an elastic tape over various body muscles. This method is used to facilitate and inhibit muscle function [1, 2], functional correction over joints or above the body part for circulation enhancement, subcutaneous lymphatic drainage, fascia adjustment, and mechanical improvement [3]. KT focuses on supporting and protecting soft body tissues without restricting their function. Furthermore, KT is considered an innovation in sports rehabilitation, commonly used to prevent sports injuries and as a treatment method in physical therapy [4, 5]. The underlying physiological effect of KT elastic tape likely involves providing physical stimulus to the skin that triggers or hinders mechanoreceptors, which may result in physiological modifications at the site of KT application [6]. A correct KT application with 25% to 50% of tension (i.e., light to moderate) is used for muscle stimulation. Applications involving greater tape tension are employed for functional correction. This technique improves sensory facilitation for either assistance or motion limitation [7, 3].

Furthermore, grip strength is an important measure of hand muscle function and is often used to predict future health issues [8, 9]. The human hand, a crucial and complex part of the upper limb, is characterized by its extensive mobility and the fine motor skills of its surrounding tissues, enabling both sensation and grip. A decrease in hand grip strength can significantly impair the ability to perform routine tasks, making the restoration of this function a key goal in rehabilitation [10]. On the biomechanical base, the grip force is created by the complex co-activation of the forearm (flexors and extensors) and hand muscles [11, 12].

Previous studies have shown different tape application methods for improving hand grip strength [7, 4, 13, 14]. In light of the previously established studies that addressed the long-term effects and application of different tapping techniques and considering the popularity of KT in the rehabilitation of hand injuries, the efficacy of KT in different population settings is needed [15]. However, the true immediate and localized effect of taping on grip strength is not yet apparent [16, 17]. This gap highlights the need for further research, particularly to investigate the immediate localized effects and the influence of varying KT tension levels on hand and forearm flexor muscles to detect the grip strength among young, sedentary women. This study focused on sedentary female volunteers for several reasons. Firstly, women are more likely to sustain injuries and stress because their muscles have not been conditioned to various activities. Secondly, the physical fitness level of sedentary participants differs from professionally trained athletes [18, 19]. Lastly, women are more likely to be exposed to cumulative trauma disorders of the upper limb, such as carpal tunnel syndrome, than men inside the workplace [20].

## MATERIAL AND METHODS

### Study design

This study used a single-blinded pre-test and post-test control group design to determine the immediate and localized effect of KT on hand grip strength in sedentary women.

### Participants and randomization

**Figure 1:**
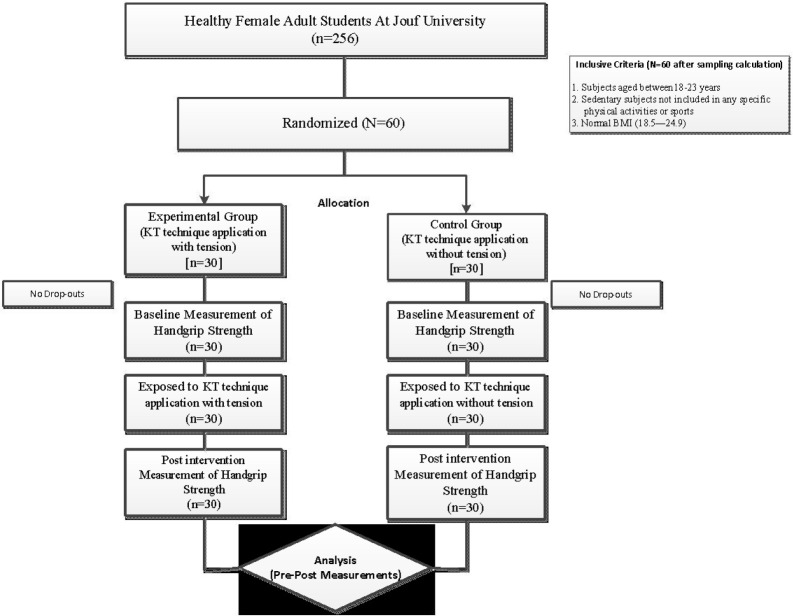
Flowchart showing randomization of participants

All healthy female adult students (N=256) from the study setting were invited to participate in this study and were subjected to selection criteria during the screening process. The required number of samples was calculated by assuming 80% power using a two-tailed test with a significance level of 0.05 and a minimum detectable change published in similar studies [21, 22]. Subsequently, at least 30 participants were needed to conduct the study, considering a 10% drop rate. Participants were selected using the following inclusion criteria: (i) women between the ages of 18 and 23; ii) subjects who had not participated in organized sports or physical activity outside of their daily routines for a minimum of six months; and (iii) subjects with a BMI between 18.5 and 24.9. Participants were excluded if they had musculoskeletal disorders, excessive body hair, fragile skin, or adhesive allergies. Based on these criteria for selection, sixty healthy women were included using an interview. The subjects were randomized to KT and control groups with a 1:1 allocation ratio, performed using computer-generated random allocation cards (RANCODE^®^, IDV, Gauting, Germany). Subjects were assigned to one of the two groups of 30 each, and the allocation concealment was carried out utilizing sealed opaque envelopes. Accordingly, thirty subjects in the experimental group received the KT technique with tension, and subjects in the control group received the placebo KT technique without tension (Figure 1).

### Blinding

Due to practical constraints and study regulations, the treating physiotherapist was not blinded. However, blinding occurred at the evaluation and data collection level, where a physical therapy assistant involved in randomization was neither involved in executing the therapeutic intervention nor the data collection process.

### Instrumentation

Body mass index and height were assessed using a measurement tape and a weight scale (Detecto 349 Height Rod Handpost Weighbeam Physician's Scale). Then, BMI was estimated by dividing the body mass (kg) by the body height squared (m^2^) using a method provided by the National Heart, Lung, and Blood Institution (U.S. Department of Health & Human Services). Furthermore, the hand grip strength was evaluated using a BASELINE^®^ Hydraulic dynamometer (NY 10602), which is certified as a gold standard tool. The consistency of this instrument is high, which makes only one measure adequate for pinch and grip strength [23]. It permits easy and rapid hand grip strength measurements, represented by pounds (lbs)/kilograms (kgs). BMI and handgrip strength were included as there is a relationship between the two variables, with both underweight and overweight participants showing lower grip strength and endurance than the normal weight group [24]. Besides, this study utilized the original Kinesio tape (Tex Classic, IC: 87683491), which is 100% cotton latex-free, hypo-allergenic, and non-restrictive. This tape has a water-resistant covering and is simple to use. It is thin and has high elasticity, allowing the tape to expand to 20-40% of its basic length. The tape is five centimeters wide and five meters long.

### Procedure

**Figure 2:**
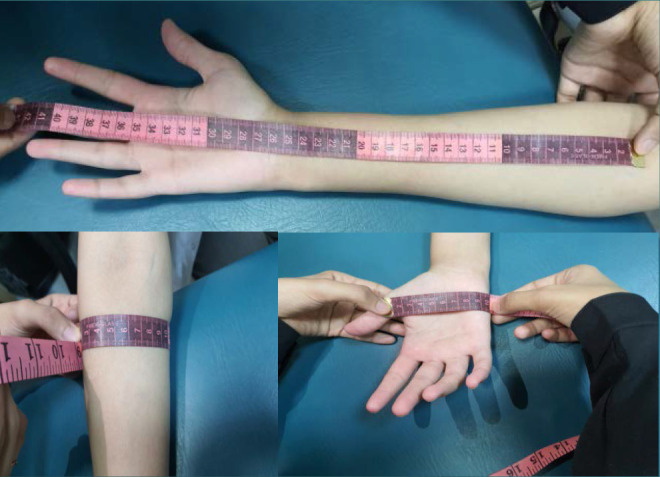
Anthropometric measurements

The study was conducted in the laboratory of Physical Therapy at the College of Applied Medical Sciences, Jouf University, between 30.06.2022 and 30.07.2022. KT tends to facilitate the primary muscles responsible for the hand grip strength (i.e., flexor digitorum superficialis muscle, flexor digitorum profundus, adductor policis musle, and flexor policis brevis muscle). The KT technique was explained to both groups, and their informed consent was obtained. Initially, anthropometric measurements such as forearm length (from the thickest part of the forearm to the hand), forearm width, and hand width were measured (Figure 2).

Furthermore, the measurement of maximum hand grip strength was performed by one examiner under the same circumstances using the hand-held dynamometer. Three trials were captured for each subject in each group (experimental and control group) at the same examination session before and after the KT technique application with tension (for the experimental group) and without tension (for the control group). Accordingly, the initial measurement of the hand grip strength of the dominant hand was carried out using the hand-held dynamometer before applying KT in both groups. Each subject was placed in a standardized position in an adjustable chair with a straight back, hips and knees flexed at 90 degrees, and feet flat on the floor. The shoulders were positioned in adduction adjacent to the trunk with the elbows in 90 degrees of flexion and the forearm and wrist in the neutral position. Also, their arms were unsupported while the investigator held the dynamometer for each reading, as recommended by the American Society of Hand Therapists (ASHT) [25]. In this position, subjects were asked to press the hand-held dynamometer with maximal strength using their dominant hand. This procedure was repeated thrice with a one-minute rest between trials. The average of three measurements was calculated as the pre-intervention hand grip strength. Subsequently, KT was applied to the dominant hand of each subject in both groups.

**Figure 3:**
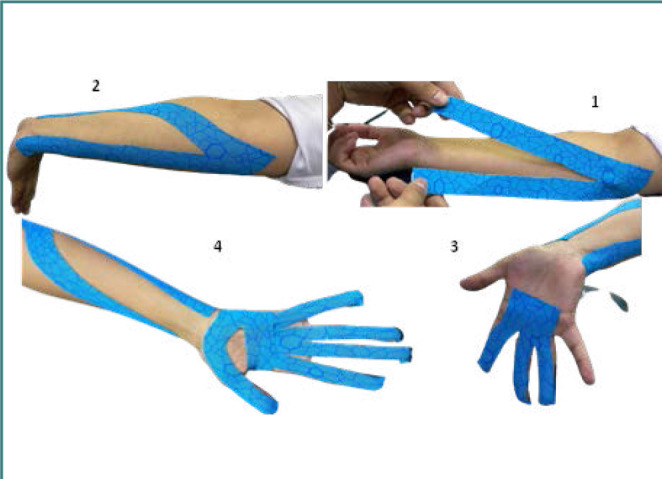
Application of Kinesio-Taping Y technique with tension

The dominant hand of each participant in the experimental group was applied with Kinesio tape. The tape was directly placed on the skin in the muscle stimulation technique, conforming to the principles of Dr. Kenso Kase. The length of the tape was detected through the distance of 2 cm below the origin of the muscle and along the length of the forearm. The tape was applied on the skin of the forearm from the flexor muscle origin to their insertion through a 50% tension of the tape to optimize the functional performance of the muscle [26]. The tape was printed with octagons in the Xact Stretch pattern. The stretch levels of the tape were indicated by small and large octagon signs at 25 and 50 percent, respectively, which are the most typical values for kinesiology tape. This creates a visual guide that the researcher can use to consistently apply kinesiology tape with the right stretch. The beginning and the end of the taping should be placed without tension. Moreover, the functional correction technique was used to stimulate finger flexion (i.e., applying the tape on fully actively extended fingers from the metacarpophalangeal joint towards the fingertips; 100% tension of the tape was applied; and no tension at both ends of the tape) [3] (Figure 3). In the control group, the dominant hand of each participant received KT as in the experimental group, but without tension (i.e., placebo intervention).

Immediately after applying KT with the remaining tape in place, the hand grip strength measurement was repeated thrice in both groups in the same standardized manner. The average of these three measurements was calculated as the post-intervention hand grip strength.

### Data analysis

The data collected on pre- and post-intervention hand grip strength were analyzed using SPSS version 20. Initial exploratory analyses were conducted to verify that the demographic data, including age, body weight, body height, body mass index (BMI), arm and forearm length, hand width, and forearm circumference, did not violate any of the assumptions related to normality, linearity, or homogeneity of variances. Also, any outliers were detected and excluded. Specifically, descriptive statistics were run to find the mean and standard deviation (SD) of each demographic variable between the control and experimental groups, such as arm and forearm length, hand width, and forearm circumference. Furthermore, inferential analysis indicated no significant differences in demographic variables between groups (P>0.05) (Table 1). Additionally, the dependent t-test was applied to assess the effect of KT application with tension (experimental group) and without tension (control group) on hand grip strength among sedentary female participants. The independent t-test was applied to determine differences in the variation of hand grip strength between KT application with tension (experimental group) and without tension (control group). In addition, the effect size (r) (Cohen's d) was calculated to assess the magnitude of change in the mean score of an outcome measure from one time point to another [27, 28]. Also, the SD approach is a distribution-based method used to calculate the minimum clinically significant difference (MCID) in hand grip strength measurement [29]. All statistical analyses were performed at a 5% significance level.

**Table 1:** Descriptive statistics of demographic data

Demographic variables	Control group (n=30) (mean ± SD)	Experimental group (n=30) (mean ± SD)	t-value	P value
**Age (years)**	19.93±1.46	20.57±1.25	**-1.085**	**0.287**
**Body height (cm)**	158.90±5.31	157.77±5.15	**0.800**	**0.431**
**Body weight (kg)**	57.37±10.66	56.63±9.10	**1.047**	**0.304**
**BMI (kg/cm^2^)**	22.94±4.08	22.72±3.63	**0.662**	**0.514**
**Length of arm and forearm (cm)**	41.3±2.2	41.6±1.8	**-0.451**	**0.655**
**Hand width (cm)**	9.5±0.52	9.3±0.46	**1.122**	**0.271**
**Forearm circumference (cm)**	24.3±1.2	23.6±1.6	**1.384**	**0.177**

Abbreviations: SD - Standard deviation; BMI - Body Mass Index. *Significant level (P<0.05)

## RESULTS

### Descriptive statistics

The Shapiro-Wilk test confirmed the homogeneity of demographic data and hand grip strength in both groups (P>0.05). We used descriptive statistics to calculate the mean and standard deviation (SD) for age, weight, height, BMI, forearm length, hand width, and forearm width. There were no statistically significant differences in the demographic data between both groups (P>0.05) (Table 1).

### Variation in hand grip strength within the group

The dependent t-test revealed no significant variation in the mean values of hand grip strength between pre- and post-intervention of KT without tension in the control group (mean difference 0.32 Lbs.; P>0.05) with no significant effect size. On the other hand, there was a significant variation in the mean values of the hand grip strength between pre-and post-intervention of KT with tension in the experimental group (mean difference of 6.5 Lbs.; 95% CI, -7.58 to -5.42; P<0.05) with a high effect size (Table 2).

**Table 2:** Dependent t-test showing variation in hand grip strength within the group (pre-test versus post-test analysis)

Variable	Group	Pre-intervention	Post-intervention	t value	95% CI of Difference		Effect Size (r)
					Lower	Upper	
**Handgrip strength (lbs)**	Experimental group	52.92±6.53	59.42±5.71	-12.35* (P=0.00)	-7.58	-5.42	0.92 (High)
	Control group	50.22±6.94	49.90±6.77	t=0.436 (P=0.666)	-1.17	1.80	0.081 (No Effect)

* Significance at 0.05 level (P<0.05)

In addition, this study observed the calculated MCID values (threshold) for handgrip strength as 0.86 and 1.44 for the control and experimental groups, respectively. Additionally, the study found that all subjects (100%) in the experimental group who received KT with tension met or exceeded the MCID threshold of 1.44 for hand grip strength, whereas only 3% of the control group achieved the 0.86 MCID values (threshold) for hand grip strength.

### Variation in hand grip strength between groups

The independent t-test indicated no significant variation in hand grip strength between KT application with tension (experimental group) and without tension (control group) (P>0.05) before intervention (Table 3). However, a significant variation was observed in hand grip strength between KT application with tension (experimental group) and without tension (control group) (P<0.05) at the post-intervention stage, where the experimental group showed greater improvement in handgrip strength (mean difference of 6.5 Lbs; 95% CI, -12.90 to -6.54; P<0.05) with a medium effect size (Table 3). This finding implies a clinically significant difference in the post-intervention mean of hand grip strength between the experimental and control groups with a medium effect size.

**Table 3:** Independent t-test showing variation in hand grip strength between groups

Variable	Intervention stage	Experimental group	Control group	t-value	95% CI of Difference		Effect Size (r)
					Lower	Upper	
**Handgrip strength (lbs)**	Pre-intervention	52.92±6.53	50.22±6.94	-1.552 (P=0.126)	-6.18	0.78	0.20 (Small Effect)
	Post-intervention	59.42±5.71	49.70±6.57	-6.12* (P=0.00)	-12.90	-6.54	0.63 (Medium)

* Significance at 0.05 level (P<0.05)

## DISCUSSION

The current study evaluated the immediate and localized effect of KT on hand grip strength in sedentary female participants. The results showed no significant improvement in hand grip strength following KT application without tension (control group), where only 3% of subjects achieved 0.86 MCID values (threshold). In contrast, a significant improvement was observed in hand grip strength following KT application, with 50% tension on the forearm and 100% on the hand (experimental group). A mean difference of 6.5 Lbs in hand grip strength was observed following KT application with tension, where 100% of participants in the experimental group achieved 1.44 MCID values (threshold). Besides, a significant increase in the mean of hand grip strength was observed in the experimental group compared to the control group at the post-intervention stage. In line with these findings, Lemos *et al*. [30] examined the variation in hand grip strength following KT intervention with or without moderate tension to the non-dominant and dominant arms. A significant improvement in hand grip strength was found in the Kinesio group compared to the control group after 24 hours for the right hand only and 48 hours for both hands. Such changes following KT might be possible since the stretch from the taping develops tension in the skin that enhances the exchange of information with mechanoreceptors and augments the motor unit recruitment throughout a muscle contraction. KT could boost the strength of weakened muscles by improving muscle performance with stimulation and reinforcement [26]. Previous studies also stated that KT enhances electromyographic activity [31-34]. The present study differs from the previous research by Lemos *et al*. [30] concerning the dominant arm only, the amount of tension applied (25-35% tension), and time points of measurement (30 minutes, 24 hours, and 48 hours). However, both studies were conducted on healthy female participants. Contrarily, a recent study investigated the immediate effect of KT with 35% tension on hand grip strength among healthy men and found an immediate improvement in hand grip strength following 30 minutes of KT. However, the control group receiving KT without tension failed to show significant improvement in hand grip strength immediately [35].

On the other hand, a previous study on healthy volunteers found a significant variation in hand grip strength among muscle facilitation, inhibition, and placebo groups of the dominant and non-dominant arms that received KT. The muscle facilitation and inhibition groups received KT with 10-15% tension, and the placebo group had KT without tension. The hand grip strength was measured 24, 48, and 72 hours after KT application. Furthermore, the results showed that the action exerted by KT peaked at 48 hours, after which it decreased, and KT failed to produce a significant late effect on hand grip strength [36]. Likewise, the current study revealed a significant difference in hand grip strength between the experimental and control groups immediately after KT application. This finding was observed immediately following the application of KT.

Furthermore, Donec *et al*. [3] performed similar research on 54 healthy subjects, where the KT group received special KT to 32 hands, and the placebo group received placebo taping to 22 hands. The KT group failed to show variations in maximum key pinch force following 30 minutes. However, it showed a significant improvement in maximum key pinch force after one hour, maximum grip force after 30 minutes, and one hour after KT application. The tension applied during KT in that research is similar to the present study. This improvement effect might be because of the reflex mechanism of the nervous system following KT application.

Furthermore, the taping on the skin continually excites cutaneous mechanoreceptors, thereby delivering additional sensory signals to the brain for information integration [37]. Earlier studies also detected augmented recruitment of motor units and enhanced bio-electrical activity directly after KT intervention [38, 32]. However, a previous study showed no significant changes in hand grip strength in the KT group compared to the control group, which included female volleyball players. In that study, the KT was placed with tension from about 10-15% in the “Y-shape” technique only in the middle part of the forearm without the application on the hand and fingers, and the sample selected was athletes [39].

Similar to our study, previous research involving healthy university students explored the effects of I-shaped forearm KT, but it differed in its approach by including both male and female participants. This study focused on identifying the optimal region for KT application and examined its effects over various time intervals - immediately after taping and then at one, one and a half, and two hours post-application. The KT was applied to both the forearm flexor and extensor muscle groups, maintaining a consistent tension of 50%. This study found that applying KT to the forearm extensor region effectively enhances grip strength [20] since the extensor muscles stabilize the grip and exert a compressive force on the wrist and metatarsophalangeal joints and provide balancing force to the forearm flexors, causing stronger contraction by preserving the length-tension relationship [40]. In addition, higher grip strength was noticed in women, though both genders showed increased grip strength at a slower pace [20].

This study was restricted to a smaller sample size of healthy female participants, which narrowed the generalization of the outcomes. Future research should include both genders and a larger sample size of healthy subjects. Additionally, the current study was designed as a single-session clinical trial involving KT application for a brief period in both experimental and control groups. Future studies could benefit from extending the duration of KT application while maintaining similar tension levels. Furthermore, there needs to be a follow-up to determine the actual effect of the current study, which must be addressed in future studies. Lastly, this study explored the effect of KT with tension on healthy subjects, and further research is warranted to reveal the effect of KT on patients' grip strength and key pinch force.

### Clinical implications

The findings from this study provide valuable insights for physical therapists (PTs), particularly in the context of sports. They suggest that PTs could effectively use the KT technique on athletes just before competitions that demand strong hand grip. Additionally, PTs use KT in their clinical practice to treat patients needing more grip strength and key pinch force to improve their hand function, especially untrained individuals who are more susceptible to stress and injuries. The study results are also helpful for sports professionals and physical education teachers to understand the immediate and localized effect of taping on grip strength.

## CONCLUSION

The present study concluded that applying the KT technique with tension on the forearm and hand had short-term effects on handgrip strength in healthy female participants. Specifically, KT techniques with a 50% tension of the tape facilitated muscle strength and encouraged finger flexion, resulting in immediate significant variations in hand grip strength. This technique encourages PTs to apply KT to those who require maximum hand grip strength while performing different activities through augmenting muscle function.

## Data Availability

The data that support the results of the current study are available from the corresponding author upon reasonable request.

## Personal thanks

The authors thank all participants who participated patiently to complete this study.
